# Ro 04-6790-induced cognitive enhancement: No effect in trace conditioning and novel object recognition procedures in adult male Wistar rats

**DOI:** 10.1016/j.pbb.2014.10.006

**Published:** 2014-12

**Authors:** K.E. Thur, A.J.D. Nelson, H.J. Cassaday

**Affiliations:** School of Psychology, University of Nottingham, Nottingham, UK

**Keywords:** Trace conditioning, Object recognition, 5-Hydroxytryptamine_6_ receptor, Ro 04-6790, Rat

## Abstract

The evidence for cognitively enhancing effects of 5-hydroxytryptamine_6_ (5-HT_6_) receptor antagonists such as Ro 04-6790 is inconsistent and seems to depend on the behavioral test variant in use. Trace conditioning holds promise as a behavioral assay for hippocampus-dependent working memory function. Accordingly, Experiment 1 assessed the effect of Ro 04-6790 (5 and 10 mg/kg i.p.) on associating a noise conditioned stimulus paired with foot shock (unconditioned stimulus) at a 3 or 30 s trace interval in adult male Wistar rats. Contextual conditioning was measured as suppression to the contextual cues provided by the experimental chambers and as suppression to a temporally extended light background stimulus which provided an experimental context. Experiment 2 assessed the effect of Ro 04-6790 (5 and 10 mg/kg i.p.) on recognition memory as tested by the exploration of novel relative to familiar objects in an open arena. In Experiment 1, Ro 04-6790 (5 and 10 mg/kg) was without effect on trace and contextual conditioning. In Experiment 2, there was no indication of the expected improvement under Ro 04-6790 at the same doses previously found to enhance recognition memory as measured in tests of novel object exploration. Thus, there was no evidence that treatment with the 5-HT_6_ receptor antagonist Ro 04-6790 acted as a cognitive enhancer in either trace conditioning or object recognition procedures. We cannot exclude the possibility that the experimental procedures used in the present study would have been sensitive to the cognitive enhancing effects of Ro 04-6790 in a different dose range, behavioral test variant, or in a different strain of rat. Nonetheless the drug treatment was not ineffective in that object exploration was reduced under 10 mg/kg Ro 04-6790.

## Highlights

• Trace conditioning was examined at a 30 s interval suitable to detect enhanced conditioning.
• Object recognition used parameters suitable to detect enhanced recognition memory.
• Ro 04-6790 was without effect on trace and contextual conditioning.
• Ro 04-6790 did not improve recognition memory.
• Ro 04-6790 decreased object exploration at 10 mg/kg.

## 1 Introduction

The serotonergic (5-hydroxytryptamine, 5-HT) system is involved in a variety of cognitive and behavioral processes, including various aspects of learning and memory (Altman and Normile, 1988, McEntee and Crook, 1991, Meneses and Pérez-Garcia, 2007). Of the multiplicity of 5-HT receptor sub-types, the recently identified 5-HT_6_ receptor has been a particular target for cognitive enhancers. 5-HT_6_ receptor expression is mainly restricted to the CNS, in particular in brain areas involved in learning and memory such as the hippocampus, frontal and entorhinal cortex (Monsma et al., 1993, Gerard et al., 1996, Gerard et al., 1997). 5-HT_6_ receptor functionality is complex (Borsini et al., 2011, Ramỉrez, 2013) and interactions with cholinergic and glutamatergic systems mean that effects on learning and memory may be indirectly mediated (Sleight et al., 1998, Kendall et al., 2011, Tassone et al., 2011, Ramỉrez, 2013). Nonetheless the therapeutic potential of 5-HT_6_ compounds is of great interest.

The 5-HT_6_ antagonist used in the present study, Ro 04-6790, has good selectivity for 5-HT_6_ relative to other receptors (Sleight et al., 1998). Moreover, antagonists such as Ro 04-6790 may have some regional selectivity to distinct neuronal populations (Fone, 2008, Meneses et al., 2011). Intriguingly, 5-HT_6_ receptor antagonists have previously been reported to have pro-cognitive effects in some (King et al., 2004, Pitsikas et al., 2008, Liu and Robichaud, 2009, Rosse and Scaffhauser, 2010, Gravius et al., 2011, Kendall et al., 2011) but not all (Lindner et al., 2003, Leng et al., 2003, Gravius et al., 2011) behavioral tests suitable to detect cognitive enhancement. However, to the authors' knowledge, their effects on trace conditioning have yet to be reported.

Trace conditioning procedures use the introduction of a time interval to manipulate associability of the conditioned stimulus (CS). Normally, a CS (e.g. noise) closely followed by a motivationally significant unconditioned stimulus (UCS, e.g. foot shock) becomes better associated than does a CS followed by a longer intervening interval to the UCS (Kamin, 1965). The ability to bridge longer trace intervals has been related to hippocampus-dependent working memory function (McEchron and Disterhoft, 1997, McEchron et al., 1998, McEchron et al., 2003, Sweatt, 2004). Trace conditioning also holds promise as a behavioral assay for age-related memory decline: it is impaired in aged rabbits (Graves and Solomon, 1985), rats (McEchron et al., 2004, Moyer and Brown, 2006) and mice (Galvez et al., 2011, Kishimoto et al., 2001), as well as in a mouse model of senescence (Lopez-Ramos et al., 2012).

The 30 s trace interval used in the present study supports very little conditioning in untreated animals and thus provides a suitable baseline from which to detect enhanced conditioning over a trace interval (Norman and Cassaday, 2003, Horsley and Cassaday, 2007, Grimond-Billa et al., 2008, Nelson et al., 2011b). Additionally, the procedure used in the present study has been adapted to include an experimental background stimulus which provides a measure of contextual conditioning (Rawlins and Tanner, 1998, Cassaday et al., 2001, Nayak and Cassaday, 2003, Norman and Cassaday, 2003, Horsley and Cassaday, 2007, Grimond-Billa et al., 2008). Contextual conditioning can also be assessed by measuring the animals' subsequent responses to the experimental chambers in which they were conditioned (Norman and Cassaday, 2003, Horsley and Cassaday, 2007, Grimond-Billa et al., 2008). Contextual conditioning is known to be hippocampus dependent (Winocur et al., 1987, Cassaday and Rawlins, 1997, McEchron et al., 1998) and provides an additional test for pro-cognitive effects. The availability of competing cues allows us to examine selectivity in associative learning using the trace conditioning procedure. The indirect catecholamine agonists d-amphetamine (Norman and Cassaday, 2003) and methylphenidate (Horsley and Cassaday, 2007) both increased associative learning to both trace conditioned and contextual cues, a profile consistent with reduced selectivity. In contrast, the neurotensin agonist PD 149163 selectively increased trace conditioning but did not affect conditioning at 0 s interval between CS and UCS, and reduced conditioning to box context, a profile consistent with increased selectivity (Grimond-Billa et al., 2008).

Recognition memory depends on the ability to discriminate a novel stimulus from one that has been encountered previously and is also central to our ability to remember. Tests of object recognition, which exploit rodents' natural tendency to preferentially explore novel objects, have been successfully used to investigate the neurobiological substrates of recognition memory (Ennaceur and Delacour, 1988, Dere et al., 2007, Winters et al., 2008, Warburton and Brown, 2010, Nelson et al., 2010, Nelson et al., 2011a). Tests of novel object recognition have the advantage that they do not require rule learning, extensive training or reinforcement and hence avoid the effects of such confounds on performance. Accordingly novel object exploration was included in the present study by way of a positive control for the effects of Ro 04-6790 (King et al., 2004, Gravius et al., 2011, Kendall et al., 2011). Novel object recognition was tested with a 4 h interval between the sample and test stages of the procedure. Under the procedural conditions adopted in the present study, this interval is generally sufficient to prevent untreated rats from discriminating between novel and familiar objects and thus a suitable baseline from which to detect enhanced recognition memory under Ro 04-6790. The specific doses of Ro 04-6790 examined were those previously found to enhance recognition memory tested after a 4 h delay (King et al., 2004, Kendall et al., 2011). Thus, the effects of Ro 04-6790 were compared in a trace conditioning procedure which holds promise as a behavioral assay for hippocampus-dependent working memory function, as well as in the standard test of object recognition memory.

## 2 Materials and methods

### 2.1 Animals

Seventy-two experimentally naïve adult (age range 6–7 weeks) male Wistar rats (Charles River, UK) were maintained in a conventional laboratory animal unit on a 12:12 h light/dark cycle with food and water ad libitum for use in Experiment 1. They were caged in pairs in standard opaque polypropylene laboratory rat cages (RB3; North Kent Plastics, UK). The cages had solid bottoms (floor space of 960 cm^2^) covered with sawdust bedding. Environmental enrichment was provided by cardboard tubing and other nesting materials. Rats were handled for approximately 5 min per day for 1 week and then at mean weight 218 g (range 203–239 g) were placed on water deprivation immediately prior to the conditioning procedures. The 24 rats injected with saline only in Experiment 1 were subsequently tested in Experiment 2 after an interval of one week, counterbalanced for their previous behavioral condition.

All procedures were carried out in accordance with the United Kingdom (UK) Animals Scientific Procedures Act 1986, Project License number PPL 40/3163, which ensures full compliance with the EU Directive 2010/63/EU for animal experiments.

### 2.2 Drugs

Ro 04-6790 (Tocris, UK) was dissolved in distilled water at 5.0 and 10.0 mg/ml for injection (i.p.) at 1 ml/kg to administer a dose of either 5.0 or 10.0 mg/kg. Control rats were injected with the equivalent volume of saline. Drug or control injections were administered 20 min prior to the conditioning stages of the procedure in Experiment 1.

In Experiment 2, Ro 04-6790 or saline was injected 20 min prior to the acclimatization session which preceded the tests of novel object exploration.

### 2.3 Experiment 1: effects of Ro 04-6790 in a trace conditioning procedure

#### 2.3.1 Behavioral conditioning apparatus

Six identical fully automated conditioning boxes, housed within sound-attenuating cases containing ventilation fans (Cambridge Cognition, Cambridge, UK), were used. The inner conditioning box walls consisted of plain steel (25 cm × 25 cm × 22 cm high) with a Plexiglas door (27 cm × 21 cm high), at the front. The floor was a shock grid with steel bars 1 cm apart and 1 cm above the lip of a 7 cm deep sawdust tray. A waterspout was mounted on one wall. The spout was 5 cm above the floor and connected to a lickometer supplied by a pump. Licks were registered by a break in the photo beam within the spout, which also triggered water delivery of 0.05 ml per lick. The waterspout was illuminated when water was available. A loudspeaker for the presentation of auditory stimuli was set in the roof. A 5 s mixed frequency noise set at 85 dB served as the CS. A continuous flashing light provided by the three wall-mounted stimulus lights and the house light flashing served as an experimental background stimulus. Based on previous studies, foot shock of 1 s duration and 1 mA intensity provided the UCS (Norman and Cassaday, 2003). This was delivered through the grid floor by a constant current shock generator (pulsed voltage: output square wave 10 ms on, 80 ms off, 370 V peak under no load conditions, MISAC Systems, Newbury, UK). Stimulus control and data collection were by an Acorn Archimedes RISC computer programmed in Basic with additional interfacing using an Arachnid extension (Cambridge Cognition).

#### 2.3.2 Behavioral conditioning procedure

Water deprivation was introduced 1 day prior to shaping. Thereafter, the animals received 1 h and 15 min of ad libitum access to water in their home cage at the same time each day, in addition to access to water in the conditioning apparatus on all the experimental days except conditioning. The stages of the trace conditioning procedure were as follows.

##### 2.3.2.1 Pre-conditioning to establish baseline lick response

In order to initiate licking behavior, rats were placed in the conditioning boxes with their respective cage mate and were shaped for 1 day until all drank from the waterspout. No data were recorded. Thereafter, animals were individually assigned to a conditioning box for the duration of the experiment (counterbalanced by experimental group).

There then followed 5 days of pre-training, in which rats drank in their conditioning boxes for 15 min each day (timed from first lick). The drinking spout was illuminated throughout, but no other stimuli were presented in this phase. Latency to first lick was recorded to assess any pre-existing differences in readiness to drink (prior to conditioning).

##### 2.3.2.2 Conditioning with foot shock

Conditioning was conducted following pre-training. No water was available within the box and the waterspout was not illuminated. A continuous flashing light was used as a background stimulus. There were 2 conditioning trials in which the UCS foot shock was delivered with either a 3 or 30 s trace interval following termination of the CS. The first pairing of CS and UCS was presented after 5 min had elapsed, and the second pairing was 5 min after the first, followed by a further 5 min left in the apparatus. In the absence of drinking, there were no behavioral measures to record.

##### 2.3.2.3 Reshaping after foot shock

On the day following conditioning, animals were reshaped following the same procedure as in pre-training sessions. This was done in order to re-establish drinking after conditioning. Additionally, the reshaping latencies provided a measure of contextual conditioning as reflected in suppression to the contextual cues provided by the experimental chambers.

##### 2.3.2.4 Conditioned suppression tests

On the day following reshaping, the animals were placed in the conditioning boxes and underwent an extinction test to the CS. Water was available throughout the test and the waterspout was illuminated. Once the animals had made 50 licks, the CS was presented for 15 min. The latency to make 50 licks in the absence of the CS (the A period, timed from the first lick made in each box) provided a measure of any individual variation in baseline lick responding. This was compared with the time taken to complete 50 licks following CS onset (B period) in a suppression ratio (A/(A + B)) to assess the level of conditioning to the CS, adjusted for any individual variation in drink rate. Conditioning to the experimental background stimulus was tested in the same way 24 h later (i.e. 48 h after reshaping).

### 2.4 Experiment 2: effects of Ro 04-6790 on object exploration

#### 2.4.1 Object recognition apparatus

All testing was conducted in a rectangular arena that was made of opaque plastic and measured 42 cm × 52 cm. The walls were 40 cm high. An overhead camera was used to record the animals' behavior for subsequent analysis.

The stimuli consisted of duplicate copies of objects (bottles and flasks) made of glass or metal, that varied in shape, color and size and were too heavy to be displaced by the animal. These objects did not appear to share common features, and had previously been found to support robust novel object recognition performance in the same arena. Pairs of objects were placed in opposite sides of the arena. The test box and objects were cleaned with an alcohol-based solution (20% w/v) before each trial to remove odor cues. The particular set of objects used was counterbalanced and at test the placement was counterbalanced between animals. The test objects were always identical copies of the object or objects seen at sampling. Animals were consistently placed in the center of the arena at the start of the sample and test sessions. Time spent exploring each object was defined as directing the nose at the object at a distance of less than 1 cm and actively exploring it (i.e. sniffing and or interacting with the object). Object exploration was not scored if the animal was in contact with but not facing the object or if it sat on the object or used it as a prop to look around or above the object (Ennaceur and Delacour, 1988, Dix and Aggleton, 1999). Animals were returned to the home cage with their respective mate in an adjoining holding area between sample and test phases.

#### 2.4.2 Object recognition test procedures

##### 2.4.2.1 Pre-test habituation

Prior to the start of testing, animals received 1 habituation session. The rats were placed individually into the arena for 10 min, the day before testing.

##### 2.4.2.2 Recognition memory testing

Ro 04-6790 or saline was administered 20 min before a further 3 min habituation session run immediately prior to the sample stage (‘acclimatization’), during which the rat was again placed in the area in the absence of objects. There was a 1 min interval between the 3 min acclimatization and the sample phase, which the animals spent with their cage mate in their home cage. During the sample phase, the animals were allowed to explore two identical copies of the sample object for a period of 3 min. The total time spent exploring the two identical objects was recorded. After a delay of 4 h, each rat was returned to the arena, which now contained a novel object and an identical copy of the object previously seen during the sampling phase. Each rat was tested once for 3 min.

Exploration of novel versus familiar objects was scored in each of the three 1-min time bins by an independent experimenter who was blind to the animals' drug group. A second independent experimenter rescored 20% of all test phases from the original video footage. Successful novel object recognition was indexed by greater exploration of the novel compared to the familiar object. The discrimination ratio was calculated as the total time spent exploring the least recently seen object divided by the time exploring both objects sampled at test.

### 2.5 Experimental design and statistical analysis

In Experiment 1, there were 6 experimental groups run in a 3 × 2 independent factorial design with drug at levels saline, 5.0 mg/kg, and 10.0 mg/kg, and behavioral condition at levels 3 or 30 s trace interval (n = 12/group). Statistical analysis was performed using analysis of variance (ANOVA). ANOVA of the pre-training latencies included the additional factor of days (at 5 levels). The dependent variables were lick latencies at pre-conditioning and reshaping, and the A periods and suppression ratios for the test of suppression to the CS and the background stimulus. Where necessary, raw latency data (time to first lick at reshape) were log transformed so that their distribution was suitable for parametric analysis.

In Experiment 2, there were 3 experimental groups with drug at levels saline, 5.0 mg/kg, and 10.0 mg/kg (n = 8/group). Planned comparison one-sample one-tailed *t*-tests were performed (with the test value set at 0.5 indicating equivalent exploration of the two objects), in order to establish whether the animals' novel object exploration was above chance. The discrimination ratio was also analyzed by one-way ANOVA with the factor of drug at levels saline, 5.0 mg/kg, and 10.0 mg/kg. Additional analyses examined drug effects on exploration in 3 × 2 mixed designs: first to examine the level of exploration at test as a function of object exposure (novel or familiar); and second to examine overall exploration of either object in relation to stage of procedure (sample or test). Additionally, repeated measures analyses compared novel versus familiar object exploration over the three 1-min time bins of test. Thus the dependent variables were the discrimination ratio, the total time spent exploring the novel object divided by the time exploring both objects sampled at test, and the (min-by-min) raw score exploration durations. No rats needed to be excluded because of a failure to explore the objects at the sample stage of the procedure. Where appropriate, differences between drug groups were explored with two-tailed independent *t*-tests.

## 3 Results

### 3.1 Experiment 1: effects of Ro 04-6790 in a trace conditioning procedure

#### 3.1.1 Pre-conditioning — baseline lick latencies

As would be expected animals drank more readily over successive exposures to the experimental chambers and this was reflected in a main effect of days statistically [*F*(4,264) = 19.323, p < 0.001]. However, there were no systematic differences in latency to lick as a function of drug or conditioning group-to-be, neither was there any interaction between these factors [maximum *F*(2,66) = 0.689, p = 0.506].

#### 3.1.2 Reshaping — conditioning effects on lick latencies

There was no effect of trace conditioning group, no effect of drug and no interaction between these factors [maximum *F*(1,66) = 1.424, p = 0.237].

#### 3.1.3 Conditioned suppression tests

**Fig. 1:**
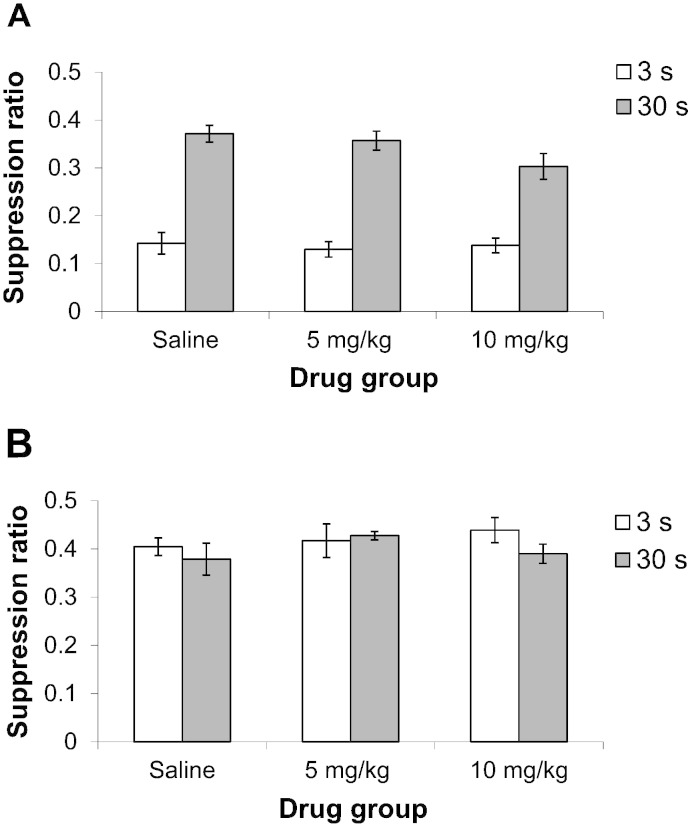
Experiment 1 trace conditioning: (A) mean suppression ratio (± S.E.M.) to a noise CS for rats conditioned at 3 s (white bars) or 30 s trace interval (dark gray bars) groups following treatment with saline, 5 or 10 mg/kg Ro 04-6790. (B) Mean suppression ratio (± S.E.M.) to the light background stimulus for rats previously conditioned at 3 s (white bars) or 30 s trace interval (dark gray bars) groups under saline, 5 or 10 mg/kg in Ro 04-6790.

Prior to the presentation of the CS, drinking during the A period was well matched. There was again no effect of drug or trace conditioning group [maximum *F*(2,66) = 1.096, p = 0.340]. On the suppression ratio measure of learning, there was a significant main effect of trace conditioning group [*F*(1,66) = 158.270, p < 0.001], reflecting an overall reduction in learning in the 30 s trace group. However, as shown in Fig. 1A, there was no effect of drug, nor any drug by trace conditioning group interaction [maximum *F*(2,66) = 1.668, p = 0.196].

Fig. 1B shows that there were no differences in suppression to the light background stimulus, either by trace or drug condition, and this was confirmed by ANOVA [maximum *F*(1,66) = 1.078, p = 0.303].

### 3.2 Experiment 2: effects of Ro 04-6790 on object exploration

The rescored results significantly correlated with the original scores (*r* = 0.985, p < 0.001, for the discrimination ratios) indicating robust inter-rater reliability.

**Fig. 2:**
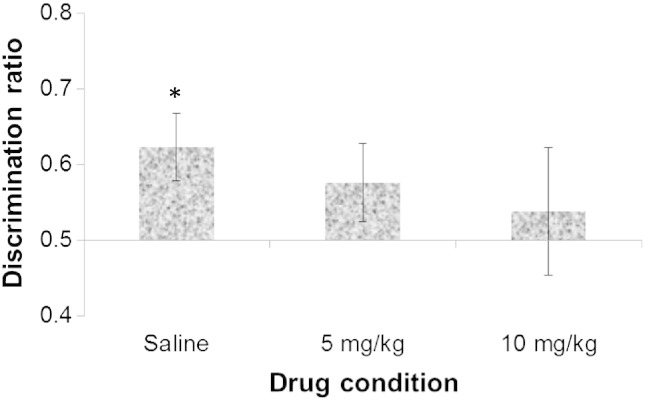
Experiment 2 novel object recognition: mean discrimination ratios (+ S.E.M.) following treatment with saline, 5 or 10 mg/kg Ro 04-6790; * denotes performance above chance, one-tailed *t*-test, p < 0.05.

The object recognition parameters had been selected to be suitable to detect enhanced recognition memory under Ro 04-6790. Fig. 2 shows that the discrimination ratios were low and planned comparison confirmed that performance was just above chance in the saline group [*t*(7) = 2.013, p = 0.042; one-tailed]. However, there was no significant main effect of drug on the discrimination ratio measure [*F*(2,21) = 0.285, p = 0.755]. Furthermore, there was no indication of improved object recognition under either dose Ro 04-6790 in that the discrimination ratios were lower than that seen in the saline group and neither the 5 mg/kg [*t*(7) = 1.142, p = 0.146; one-tailed] nor the 10 mg/kg Ro 04-6790 group performed above chance [*t*(7) = 0.372, p = 0.361; one-tailed].

**Fig. 3:**
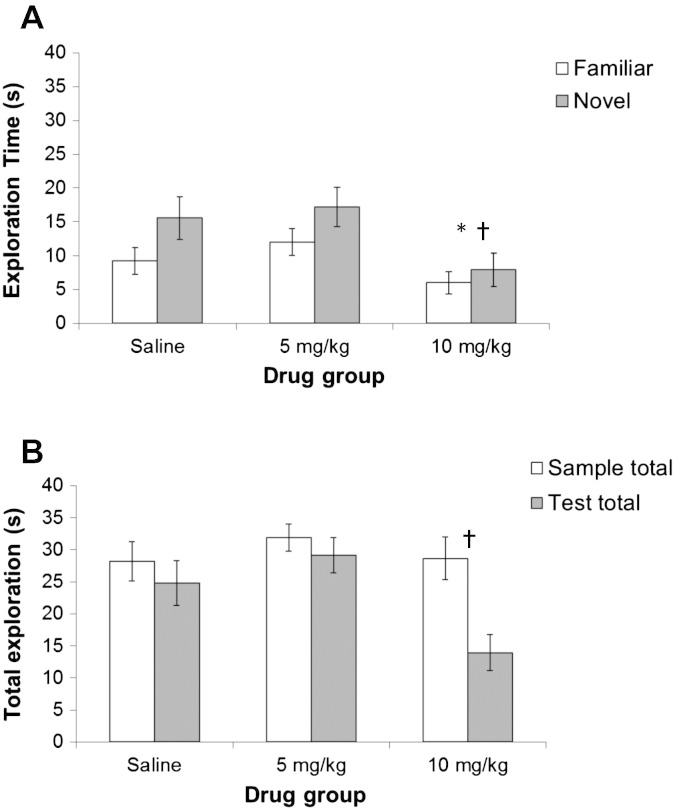
Experiment 2 novel object recognition: (A) test exploration (± S.E.M.) of familiar (white bars) versus novel object (gray bars) groups following treatment with saline, 5 or 10 mg/kg Ro 04-6790; * denotes significant difference in overall exploration relative to the saline group, *t*-test, p < 0.05; ✝ denotes significant difference in overall exploration relative to the 5 mg/kg group, *t*-test, p < 0.01. (B) Total exploration (± S.E.M.) at the sample (light gray bars) versus the test stage (dark gray bars) groups following treatment with saline, 5 or 10 mg/kg Ro 04-6790; ✝ denotes significant difference in overall exploration relative to 5 mg/kg group, *t*-test, p = 0.01.

Like Fig. 2, Fig. 3A suggests some apparent reduction in novel object recognition at 10 mg/kg Ro 04-6790. ANOVA of the test exploration times showed a marginal overall effect of object familiarity [*F*(1,21) = 4.149, p = 0.054] but in the absence of any significant interaction with drug [*F*(2,21) = 0.369, p = 0.696]. However, there was a clear main effect of drug [*F*(2,21) = 6.681, p = 0.006], consistent with some non-specific effect on exploration time under drug, rather than on the ability to recognize familiar and preferentially explore novel objects.

Post-hoc comparisons showed that the main effect of drug on test exploration time arose because test exploration was reduced under 10 mg/kg Ro 04-6790, relative to the saline condition [*t*(14) = 2.424, p = 0.029]. Moreover, test exploration was higher in the 5 mg/kg than the 10 mg/kg drug-treated group [*t*(14) = 3.885, p = 0.002]. However, any possible increase in test exploration under 5 mg/kg Ro 04-6790 was not significant compared with that seen in the saline group [*t*(14) = 0.989, p = 0.339].

Preferential exploration of the novel object can decline over a 3 min test session. Accordingly, exploration was also examined in three 1-min time bins. Although the effect of time bins was significant [*F*(2,42) = 7.556, p = 0.002], there were no significant interactions between time bins and familiarity or drug [maximum *F*(4,42) = 1.532, p = 0.210, for the three-way interaction].

Fig. 3B shows the total exploration times as a function of (sample versus test) stage of procedure. As might be expected, animals showed reduced overall exploration at test compared with that seen at the sample stage [*F*(1,21) = 9.676, p = 0.005]. ANOVA of the total exploration times also showed an overall effect of drug [*F*(2,21) = 4.257, p = 0.028] in the absence of any significant interaction with stage of procedure [*F*(2,21) = 3.026, p = 0.070]. Post-hoc comparisons showed that the main effect of drug on exploration time arose because this was overall lower in the 10 mg/kg than the 5 mg/kg drug-treated group [*t*(14) = 2.936, p = 0.011]. Thus, there were also dose-related effects on overall exploration. However, any increase in overall exploration under 5 mg/kg Ro 04-6790 was not significant compared with that seen in the saline group [*t*(14) = 1.403, p = 0.182] and there was no evidence that overall exploration was reduced under 10 mg/kg Ro 04-6790, relative to the saline condition [*t*(14) = 1.498, p = 0.156].

## 4 Discussion

In Experiment 1, there was no evidence that treatment with the 5-HT_6_ receptor antagonist Ro 04-6790 had pro-cognitive effects in the trace conditioning procedure. Both lesion (McEchron et al., 1998) and electrophysiological studies of the hippocampus (McEchron and Disterhoft, 1997, McEchron et al., 2003, Gilmartin and McEchron, 2005) have confirmed its role in trace conditioning using procedures comparable to those used in the present study. Specifically, impaired trace conditioning has been attributed to impaired hippocampus-dependent working memory function (Sweatt, 2004). Conversely, enhanced conditioning over a 30 s trace interval has been demonstrated after a variety of experimental treatments including d-amphetamine (Norman and Cassaday, 2003), methylphenidate (Horsley and Cassaday, 2007), the neurotensin agonist PD 149163 (Grimond-Billa et al., 2008) and catecholaminergic depletion within nucleus accumbens core (Nelson et al., 2011b). Contrary to expectation, there was no indication of any increase in conditioning over the 3 or 30 s trace interval under Ro 04-6790, the difference between groups conditioned at these intervals was statistically robust and very similar to that seen in the saline-injected controls. Nonetheless, the profile of conditioning to the other available cues should also be considered. Enhanced conditioning to contextual cues using the procedure adopted for the present study has been demonstrated after d-amphetamine (Norman and Cassaday, 2003), methylphenidate (Horsley and Cassaday, 2007) and central serotonergic depletion (Cassaday et al., 2001). However mediated, enhanced contextual conditioning could be attributed to enhanced hippocampus-dependent working memory function (Winocur et al., 1987, Cassaday and Rawlins, 1997, McEchron et al., 1998). But despite the demonstrated sensitivity of the experimental parameters in use, in the present study there was no evidence for the modulation of the level of contextual conditioning under Ro 04-6790.

We cannot exclude the possibility that the experimental test procedures used in the present study would have been sensitive to the cognitive enhancing effects of Ro 04-6790 over different trace intervals or in a different dose range. However, the 30 s trace interval used in this procedure has demonstrated sensitivity to such effects (Cassaday et al., 2001, Norman and Cassaday, 2003, Grimond-Billa et al., 2008, Horsley and Cassaday, 2007, Nelson et al., 2011b). With respect to the dose range examined, this is generally less of a concern in studies of antagonists but it must be acknowledged that 5-HT_6_ receptor functionality is complex. For example, agonists and antagonists show unexpected similarities in some of their pharmacological properties (Borsini et al., 2011) and have similar effects in some behavioral tests of pro-cognitive efficacy (Fone, 2008, Meneses et al., 2011).

Ro 04-6790 was not ineffective at the doses examined in the present study. In Experiment 2, counter to prediction (King et al., 2004, Kendall et al., 2011), rats' spontaneous preference to explore a novel object was apparently reduced by treatment with Ro 04-6790 at 10 mg/kg (Fig. 2, Fig. 3A). This reduction in exploration was not selective to the novel object in that statistically there were no interactions between drug and object familiarity. Preference for the novel object of course relies on adequate levels of exploration, particularly at the sample stage of the procedure, of the object designated the familiar alternative. Fig. 3B shows that the levels of sample exploration were closely similar in all drug conditions but – albeit the interaction between drug and stage of procedure did not reach significance – there was a greater drop in test exploration under 10 mg/kg Ro 04-6790. Since there was a dose-related effect on test as well overall exploration, any reduction in novel object exploration was most likely attributable to secondary to sedative effects of Ro 04-6790 at 10 mg/kg (Bentley et al., 1999). Sedative effects might be expected to be more apparent on the second exposure to objects in the arena, albeit over 4 h after drug administration, when the rats were further habituated. Lower dose treatment with the 5-HT_6_ antagonist Ro-4368554 has previously been reported to increase locomotor activity as measured in the open field (Gravius et al., 2011). However, whilst there was no sedation at the lower dose, there was no evidence for increased activity at 5 mg/kg under the experimental conditions of the present study. In any event, the apparent reduction in novel object recognition after 10 mg/kg Ro 04-6790 was not supported statistically in that there was no main effect of drug on the discrimination ratios and no drug by object familiarity interaction on analysis of the raw score exploration durations.

It remains possible that the demonstration of pro-cognitive versus non-specific effects might require different levels of receptor occupancy in different brain regions. Specifically, the non-specific effect on object exploration might relate to a generally sedative effect on locomotor activity, requiring a different level of receptor occupancy than would be necessary to demonstrate enhanced novel object recognition under the experimental conditions of the present study. 5-HT_6_ receptors are found in the striatum as well as the hippocampus, frontal and entorhinal cortex and there may be regional variation in the level of receptor occupancy necessary to modulate different behaviors. In any event, identifying the specific intracellular pathways modulated subsequent to receptor occupancy has been identified as key to understanding the necessary mechanisms for pro-cognitive effects mediated the 5-HT_6_ receptor (Borsini et al., 2011, Ramỉrez, 2013). Nonetheless Experiment 2 provides a positive control in that decreased object exploration – albeit irrespective of object novelty and inconsistent with any pro-cognitive effect in the present study – means that the drug treatments used in Experiment 1 were unlikely to have been generally ineffective.

The 4 h delay interval between sample and test adopted in the present study is generally sufficient to induce natural forgetting and – as expected – this was reflected in the modest discrimination ratios seen in vehicle-treated rats. In the present study, conducted in a somewhat larger arena (42 × 52 cm) than that used previously (38 × 40 cm) there was more marked forgetting in that (as shown in Fig. 2) the discrimination ratios in saline-injected animals were around 0.6 and only just above chance level performance. However despite parameters with the potential to detect enhancement in that the baseline performance in untreated animals was not high, the expected improvement in novel object recognition (King et al., 2004, Gravius et al., 2011, Kendall et al., 2011) was not demonstrated in the present study.

It must be acknowledged that there were some differences between the procedures adopted in the present versus the previous studies which showed improved recognition memory after treatment with 5HT_6_ antagonists. For example, in addition to the 1267.5 cm^2^ larger size of arena and more salient objects – bottles and flasks made of glass or metal, that varied in shape, color and size – used in the present study, there were differences in the duration of pre-test habituation which was 10 min in the present study compared with 60 min in some of the previous studies (King et al., 2004, Kendall et al., 2011). We have previously published the results of a number of novel object studies which used a 10 min habituation session (Nelson et al., 2010, Nelson et al., 2011a, Nelson et al., 2011b); in these studies in the absence of a further 3 min habituation directly prior to the sample stage. Habituation and anxiety-related effects might still confound interpretation of the results of the present study (Ennaceur, 2010), but if the apparent effect of drug was habituation-related it should interact with stage of procedure. Specifically, neophobia would be expected to be more apparent at the sample stage of the procedure when both objects are unfamiliar and there has been relatively less exposure to the test arena. However, this was not the pattern of results observed (as shown in Fig. 3B). Neither was there any indication of any effects of Ro 04-6790 on fear conditioning (measured after treatment with the same doses in Experiment 1). Rats treated with Ro 04-6790 showed the same level of fear conditioning to the contextual cues provided by the experimental chambers and there was no significant effect of drug on cue conditioning at either of the trace intervals tested. Therefore the results of Experiment 1 do not suggest any increased emotionality under Ro 04-6790.

Additionally, the present study used Wistar rats, for comparison with earlier demonstrated effects on trace conditioning (Norman and Cassaday, 2003, Horsley and Cassaday, 2007, Grimond-Billa et al., 2008) whereas previous studies (King et al., 2004, Kendall et al., 2011) were conducted using a different strain of rats — Lister hooded rather than Wistar. Similar to the present study, Ro 04-6790 at 3 and 10 mg/kg was similarly without intrinsic effect on object recognition tests in normal adult Wistar rats (Pitsikas et al., 2008). Age may also be an issue: the rats used in the present study were within the same relatively young age range as rats tested in a number of other studies of trace conditioning using the same procedures (and which have shown positive effects of drug). The effects of Ro 04-6790 might yet be demonstrated in aged rats; alternatively, following some experimental perturbation to impair task performance. A further potential limitation arises in that Experiment 2 of the present study was conducted using only the 24 saline treated rats from Experiment 1 and thus may have been underpowered. This, however, is an unlikely explanation, as both 8 (Pitsikas et al., 2008) and 12 rats per group were used in earlier drug studies with Ro 04-6790 (King et al., 2004, Kendall et al., 2011).

The present study did not compare performance in different strains or ages of rat and did not include the use of scopolamine challenge, and even with such further tests it cannot be firmly concluded that Ro 04-6790 has no therapeutic efficacy (Lindner et al., 2003). Nevertheless, if strain of rat and minor differences in experimental procedure are sufficient to account for the discrepancy between the current results and previous reports of enhanced object recognition under Ro 04-6790, this does raise questions about the reliability of Ro 04-6790 as a pro-cognitive drug (Lindner et al., 2003).

## 5 Conclusion

We found no evidence that treatment with the 5-HT_6_ receptor antagonist Ro 04-6790 acted as a cognitive enhancer in either experimental procedure. Nonetheless the drug treatment was not ineffective in that object exploration was reduced under 10 mg/kg Ro 04-6790. Decreased object exploration in an open arena could be consistent with increased emotionality. Consistent with the latter possibility, there is evidence to suggest that the 5-HT_6_ site moderates emotional learning (Yoshioka et al., 1998, Mitchell and Neumaier, 2008) and negative mood states (Hirano et al., 2009). However, in Experiment 1 of the present study there was no indication whatsoever of any effect of Ro 04-6790 on any parameter of associative learning with foot shock UCS.

## References

[bb0005] Altman H.J., Normile H.J. (1988). What is the nature of the role of the serotonergic nervous system in learning and memory: prospects for development of an effective treatment strategy for senile dementia. Neurobiol Aging.

[bb0010] Bentley J.C., Bourson A., Boess F.G., Fone K.C.F., Marsden C.A., Petit N. (1999). Investigation of stretching behaviour induced by the selective 5-HT_6_ receptor antagonist, Ro 04-6790, in rats. Br J Pharmacol.

[bb0015] Borsini F., Bordi F., Riccioni T. (2011). 5-HT_6_ pharmacology inconsistencies. Pharmacol Biochem Behav.

[bb0020] Cassaday H.J., Rawlins J.N.P. (1997). The hippocampus, objects and their contexts. Behav Neurosci.

[bb0025] Cassaday H.J., Shilliam C.S., Marsden C.A. (2001). Serotonergic depletion increases conditioned suppression to background stimuli in the rat. J Psychopharmacol.

[bb0030] Dere E., Huston J.P., De Souza Silva M.A. (2007). The pharmacology, neuroanatomy and neurogenetics of one-trial object recognition in rodents. Neurosci Biobehav Rev.

[bb0035] Dix S.L., Aggleton J.P. (1999). Extending the spontaneous preference test of recognition: evidence of object-location and object-context recognition. Behav Brain Res.

[bb0040] Ennaceur A. (2010). One-trial object recognition in rats and mice: methodological and theoretical issues. Behav Brain Res.

[bb0045] Ennaceur A., Delacour J. (1988). A new one-trial test for neurobiological studies of memory in rats. 1: Behavioral data. Behav Brain Res.

[bb0050] Fone K.C.F. (2008). An update on the role of the 5-hydroxytryptamine_6_ receptor in cognitive function. Neuropharmacology.

[bb0055] Galvez R., Cua S., Disterhoft J.F. (2011). Age-related deficits in a forebrain-dependent task, trace-eyeblink conditioning. Neurobiol Aging.

[bb0060] Gerard C., El Mestikawy S., Lebrand C., Adrien J., Ruat M., Traiffort E. (1996). Quantitative RT-RCR distribution of serotonin 5-HT_6_ receptor mRNA in the central nervous system of control or 5,7-dihydroxytryptamine-treated rats. Synapse.

[bb0065] Gerard C., Martres M.P., Lefevre K., Miquel M.C., Verge D., Lanfumey L. (1997). Immuno-localization of serotonin 5-HT6 receptor-like material in the rat central nervous system. Brain Res.

[bb0070] Gilmartin M.R., McEchron M.D. (2005). Single neurons in the medial prefrontal cortex of the rat exhibit tonic and phasic coding during trace fear conditioning. Behav Neurosci.

[bb0075] Graves C.A., Solomon P.R. (1985). Age-related disruption of trace but not delay classical conditioning of the rabbit's nictitating membrane response. Behav Neurosci.

[bb0080] Gravius A., Laszy J., Pietraszek M., Sághy K., Nagel J., Chambon C. (2011). Effects of 5-HT_6_ antagonists, Ro-4368554 and SB-258585, in tests used for the detection of cognitive enhancement and antipsychotic-like activity. Behav Pharmacol.

[bb0085] Grimond-Billa S.K., Norman C., Bennett G.W., Cassaday H.J. (2008). Selectively increased trace conditioning under the neurotensin agonist PD 149163 in an aversive procedure in which SR 142948A was without intrinsic effect. J Psychopharmacol.

[bb0090] Hirano K., Piers T.M., Searle K.L., Miller N.D., Rutter R., Chapman P.F. (2009). Procognitive 5-HT_6_ antagonists in the rat forced swimming test: potential therapeutic utility in mood disorders associated with Alzheimer's disease. Life Sci.

[bb0095] Horsley R.R., Cassaday H.J. (2007). Methylphenidate can reduce selectivity in associative learning in an aversive trace conditioning task. J Psychopharmacol.

[bb0100] Kamin L.J., Prokasy W.F. (1965). Classical conditioning: a symposium.

[bb0105] Kendall I., Slotten H.A., Codony X., Burgueño J., Pauwels P.J., Vela J.M. (2011). E-6801, a 5-HT_6_ receptor agonist, improves recognition memory by combined modulation of cholinergic and glutamatergic neurotransmission in the rat. Psychopharmacology (Berlin).

[bb0110] Kishimoto Y., Suzuki M., Kawahara S., Kirino Y. (2001). Age-dependent impairment of delay and trace eyeblink conditioning in mice. Neuroreport.

[bb0115] King M.V., Sleight A.J., Woolley M.L., Topham I.A., Marsden C.A., Fone K.C.F. (2004). 5-HT_6_ receptor antagonists reverse delay-dependent deficits in novel object discrimination by enhancing consolidation — an effect sensitive to NMDA receptor antagonism. Neuropharmacology.

[bb0120] Leng A., Ouagazzal A., Feldon J., Higgins G.A. (2003). Effect of the 5-HT6 receptor antagonists Ro 04-6790 and Ro 65-7199 on latent inhibition and prepulse inhibition in the rat: comparison to clozapine. Pharmacol Biochem Behav.

[bb0125] Lindner M.D., Hodges D.B., Hogan J.B., Orie A.F., Corsa J.A., Barten D.M. (2003). An assessment of the effects of serotonin 6 (5-HT6) receptor antagonists in rodent models of learning. JPET.

[bb0130] Liu K.G., Robichaud A.J. (2009). 5-HT_6_ antagonists as potential treatment for cognitive dysfunction. Drug Dev Res.

[bb0135] Lopez-Ramos J.C., Jurado-Parras M.T., Sanfeliu C., Acuna-Castroviejo D., Delgado-Garcia J.M. (2012). Learning capabilities and CA1-prefrontal synaptic plasticity in a mice model of accelerated senescence. Neurobiol Aging.

[bb0140] Meneses A., Pérez-Garcia G. (2007). 5-HT1A receptors and memory. Neurosci Biobehav Rev.

[bb0145] Meneses A., Pérez-Garcia G., Ponce-Lopez T., Castillo C., Borsini F. (2011). Pharmacology of 5-HT_6_ receptors, part II.

[bb0150] McEchron M.D., Bouwmeester H., Tseng W., Weiss C., Disterhoft J.F. (1998). Hippocampectomy disrupts auditory cued fear conditioning and contextual fear conditioning in the rat. Hippocampus.

[bb0155] McEchron M.D., Disterhoft J.F. (1997). Sequence of single neuron changes in CA1 hippocampus of rabbits during acquisition of trace eyeblink conditioned responses. J Neurophysiol.

[bb0160] McEchron M.D., Tseng W., Disterhoft J.F. (2003). Single neurons in CA1 hippocampus encode trace interval duration during trace heart rate (fear) conditioning in rabbit. J Neurosci.

[bb0170] McEchron M.D., Cheng A.Y., Gilmartin M.R. (2004). Trace fear conditioning is reduced in the aging rat. Neurobiol Learn Mem.

[bb0175] McEntee W.J., Crook T.H. (1991). Serotonin, memory, and the aging brain. Psychopharmacology (Berlin).

[bb0180] Mitchell E.S., Neumaier J.F. (2008). 5-HT_6_ receptor antagonist reversal of emotional learning and prepulse inhibition deficits induced by apomorphine or scopolamine. Pharmacol Biochem Behav.

[bb0185] Monsma F.J., Shen Y., Ward R.P., Hamblin M.W., Sibley D.R. (1993). Cloning and expression of a novel serotonin receptor with high affinity for tricyclic psychotropic drugs. Mol Pharmacol.

[bb0190] Moyer J.R., Brown T.H. (2006). Impaired trace and contextual fear conditioning in aged rats. Behav Neurosci.

[bb0195] Nayak S., Cassaday H.J. (2003). The novel dopamine D4 receptor agonist (PD 168,077 maleate): doses with different effects on locomotor activity are without effect in classical conditioning. Prog Neuro-Psychopharmacol Biol Psychiatry.

[bb0200] Nelson A.J.D., Thur K.E., Marsden C.A., Cassaday H.J. (2010). Dissociable roles of dopamine within the core and medial shell of the nucleus accumbens in memory for objects and place. Behav Neurosci.

[bb0205] Nelson A.J.D., Cooper M.T., Thur K.E., Marsden C.A., Cassaday H.J. (2011). The effect of catecholaminergic depletion within the prelimbic and infralimbic medial prefrontal cortex on recognition memory for recency, location and objects. Behav Neurosci.

[bb0210] Nelson A.J.D., Thur K.E., Spicer C., Marsden C.A., Cassaday H.J. (2011). Catecholaminergic depletion in nucleus accumbens enhances trace conditioning. Adv Med Sci.

[bb0215] Norman C., Cassaday H.J. (2003). Amphetamine increases aversive conditioning to diffuse contextual stimuli and to a discrete trace stimulus when conditioned at higher footshock intensity. J Psychopharmacol.

[bb0220] Pitsikas N., Zisopoulou S., Pappas I., Sakellaridis N. (2008). The selective 5-HT_6_ receptor antagonist Ro 04-6790 attenuates psychotomimetic effects of the NMDA receptor antagonist MK-801. Behav Brain Res.

[bb0225] Ramỉrez M.J. (2013). 5-HT_6_ receptors and Alzheimer's disease. Alzheimers Res Ther.

[bb0230] Rawlins J.N.P., Tanner J. (1998). The effects of hippocampal aspiration lesions on conditioning to the CS and to a background stimulus in trace conditioned suppression. Behav Brain Res.

[bb0235] Rosse G., Scaffhauser H. (2010). 5-HT_6_ receptor antagonists as potential therapeutics for cognitive impairment. Curr Top Med Chem.

[bb0240] Sleight A.J., Boess F.G., Bos M., Levet-Trafit B., Riemer C., Bourson A. (1998). Characterisation of Ro 04-6790 and Ron 63-0563: potent and selective antagonists at human and rat 5-HT_6_ receptors. Br J Pharmacol.

[bb0245] Sweatt J.D. (2004). Hippocampal function in cognition. Psychopharmacology (Berlin).

[bb0250] Tassone A., Madeo G., Schirinzi T., Vita D., Puglisi F., Ponterio G. (2011). Activation of 5-HT6 receptors inhibits corticostriatal glutamatergic transmission. Neuropharmacology.

[bb0255] Warburton E.C., Brown M.W. (2010). Findings from animals concerning when interactions between perirhinal cortex, hippocampus and medial prefrontal cortex are necessary for recognition memory. Neuropsychologia.

[bb0260] Winocur G., Rawlins J.N.P., Gray J.A. (1987). The hippocampus and conditioning to contextual cues. Behav Neurosci.

[bb0265] Winters B.D., Saksida L.M., Bussey T.J. (2008). Object recognition memory: neurobiological mechanisms of encoding, consolidation and retrieval. Neurosci Biobehav Rev.

[bb0270] Yoshioka M., Matsumoto M., Togashi H., Mori K., Saito H. (1998). Central distribution and function of 5-HT_6_ receptor subtype in the rat brain. Life Sci.

